# Enhanced Disc Herniation Classification Using Grey Wolf Optimization Based on Hybrid Feature Extraction and Deep Learning Methods

**DOI:** 10.3390/tomography11010001

**Published:** 2024-12-26

**Authors:** Yasemin Sarı, Nesrin Aydın Atasoy

**Affiliations:** 1 The Institute of Graduate Programs, Karabük University, Karabük 78050, Türkiye; yaseminsari@eskisehir.edu.tr; 2 Department of Computer Engineering, Faculty of Engineering, Karabük University, Karabük 78050, Türkiye

**Keywords:** support vector machines, ResNet50, grey wolf optimization, disc herniation, capsule networks

## Abstract

Due to the increasing number of people working at computers in professional settings, the incidence of lumbar disc herniation is increasing. Background/Objectives: The early diagnosis and treatment of lumbar disc herniation is much more likely to yield favorable results, allowing the hernia to be treated before it develops further. The aim of this study was to classify lumbar disc herniations in a computer-aided, fully automated manner using magnetic resonance images (MRIs). Methods: This study presents a hybrid method integrating residual network (ResNet50), grey wolf optimization (GWO), and machine learning classifiers such as multi-layer perceptron (MLP) and support vector machine (SVM) to improve classification performance. The proposed approach begins with feature extraction using ResNet50, a deep convolutional neural network known for its robust feature representation capabilities. ResNet50’s residual connections allow for effective training and high-quality feature extraction from input images. Following feature extraction, the GWO algorithm, inspired by the social hierarchy and hunting behavior of grey wolves, is employed to optimize the feature set by selecting the most relevant features. Finally, the optimized feature set is fed into machine learning classifiers (MLP and SVM) for classification. The use of various activation functions (e.g., ReLU, identity, logistic, and tanh) in MLP and various kernel functions (e.g., linear, rbf, sigmoid, and polynomial) in SVM allows for a thorough evaluation of the classifiers’ performance. Results: The proposed methodology demonstrates significant improvements in metrics such as accuracy, precision, recall, and F1 score, outperforming traditional approaches in several cases. These results highlight the effectiveness of combining deep learning-based feature extraction with optimization and machine learning classifiers. Conclusions: Compared to other methods, such as capsule networks (CapsNet), EfficientNetB6, and DenseNet169, the proposed ResNet50-GWO-SVM approach achieved superior performance across all metrics, including accuracy, precision, recall, and F1 score, demonstrating its robustness and effectiveness in classification tasks.

## 1. Introduction

One of the most common intervertebral disc disorders (IDDs) is lumbar disc herniation, which causes back pain and limitation of motion, although the exact cause is unknown. More than 90% of surgical spine operations are the result of an IDD [1]. Back pain is usually diagnosed by a radiologist or orthopedist through manual examination and magnetic resonance imaging (MRI) scans of the spine. The part of the spine from L1 to L5 is called the lower back [2]. A “herniated disc” is a condition caused by a rupture of the nucleus of the disc in the fibrous annulus. This gel substance stimulates spinal neurons, causing mechanical and chemical irritation, which leads to swelling and inflammation of the spinal nerves [3]. Herniated discs most commonly develop where the spinal nerves split between the lumbar vertebrae and reconnect to form the sciatic and femoral nerves, which run along the front and back of the thigh and leg, respectively [4].

Technological advancements in the medical field aim to facilitate the work of specialists, particularly those addressing lumbar region disorders, which are increasingly prevalent. Since time is a critical factor in healthcare, studies that expedite the diagnosis and treatment process are highly desirable to support medical experts.

Deep learning [2] algorithms have been applied in almost every aspect of our lives in recent years, with the aim of making our lives easier. These algorithms are particularly useful in the context of medical image segmentation and classification. A review of the literature in this field shows that deep learning is widely used in medical fields [5,6,7]. As work on the computer is predominant in daily business life at present, significant discomfort in the lumbar and neck areas can occur due to continuous sitting. Although there have been studies [8,9,10,11] on the classification of lumbar disc herniation, which is one of the most common diseases among these disorders, this study aims to contribute to the field by proposing a new method. Some studies focused on hernia or tumor classification in the medical field are discussed below.

Several studies using the same dataset stand out in the literature. For example, Adibatti et al. [12] optimized the weights of SegNet with the adaptive rain optimization algorithm (AROA) using a dataset of 515 patients and achieved 98.5% accuracy in the classification of intervertebral disc diseases with the capsule stacked autoencoder (CSAE) deep learning model When the authors compared convolutional neural network (CNN), recurrent neural network (RNN), stacked autoencoder (SAE), and capsule network (CapsNet) models, they found that CapsNets had the best results.

Similarly, in a study [9] using the DenseNet201 model with features extracted from lumbar spine MRI images, it was observed that the support vector machine (SVM) algorithm gave the best results, achieving F1 scores close to 95% in L3/L4, L4/L5, and L5/S1 classes. In another study [8], the InVGG model was used to classify lumbar diseases, achieving 96% accuracy. Prisilla et al. [10] used YOLO series models (YOLOv5, YOLOv6, YOLOv7) in their study, and reported that the YOLOv5 model stood out with over 90% accuracy for the detection of lumbar disc herniation.

Studies combining machine learning and optimization algorithms for classification in the healtcare field are also noteworthy. Vankdothu et al. [13] applied the adaptive neuro-fuzzy inference system (ANFIS) and SVM algorithms to classify images with and without brain tumors, demonstrating that hybrid techniques improve the obtained results through combining a genetic algorithm (GA) with the grey wolf optimization (GWO) and social spider optimization (SSO) methods. Another study integrating machine learning with optimization algorithms involves the classification model developed by Jia et al. [14], who integrated the bat algorithm (BDGBA) with SVM on a dataset of 780 individuals diagnosed with lumbar disc herniation.

Reddy and Gurrala [15] achieved remarkable results in diabetic retinopathy (DR) and diabetic macular edema (DME) classification using a ResNet50-based deep learning architecture. In their study, disease-specific features were extracted using attention modules, and successful results were obtained despite the small size of the used datasets. These findings influenced the choice of ResNet50-based deep learning models in our study.

Shinde et al. [16] proposed a semi-automatic method for intervertebral disc degeneration diagnosis using local binary patterns and a pre-trained CNN. An accuracy of 80.40% was achieved in classification with the multi-class SVM algorithm, and it was stated that data augmentation and the use of more advanced deep learning algorithms could yield better results.

Unal et al. [17] developed a pre-processing method called mean shift clustering-based attribute weighting (MSCBAW), tested it on two datasets, and observed significant increases in classification accuracy with the k-nearest neighbor (k-NN), radial basis function (RBF), and SVM algorithms.

Ebrahimzadeh et al. [18] used a dataset of normal and patient images for lumbar disc herniation detection and combined multi-layer perceptron (MLP), k-NN, and SVM methods with pre-processing techniques for region of interest (ROI) extraction and classification. According to the Precision values, the SVM achieved the highest accuracy, followed by MLP and k-NN.

Šušteršič et al. [19] used logistic regression, decision tree, and random forest methods for the diagnosis of lumbar disc herniation and performed classification in the pre- and post-treatment stages. In particular, the SVM algorithm stood out as a method that gave good results.

In this study, a new method is proposed to supplement the methods previously used for classification. The contributions of the proposed model are as follows:

- This method optimizes the features obtained from the fully connected (FC) layer of the ResNet50 model with GWO and classifies them using an MLP or SVM. In this way, meaningful inputs are obtained through reducing and optimizing the complex features of big data into features.
- As it is known that the training time of CapsNet is naturally longer, when compared to traditional deep learning models [20], the training time of the proposed method is shorter. The proposed method demonstrates superior performance, in terms of accuracy, F1 score, precision, and recall when compared to various deep learning and transfer learning approaches, including CapsNet, DenseNet169, EfficientNetB6, and ResNet50.
- Early and rapid detection of herniated discs plays a key role in the earlier diagnosis and treatment of this increasingly common condition. In this regard, the proposed system can classify herniated discs quickly and with high accuracy.
- Transfer learning methods are known to provide good results, in terms of accuracy, as they are pre-trained; with the proposed method, these rates are further improved.

The main motivation of this study is to help experts in the diagnosis and treatment of intervertebral disc diseases, which continue to increase in both the healthcare field and in our daily lives. By utilizing the capabilities of artificial intelligence, deep learning, machine learning algorithms, and optimization methods, we aim to create highly accurate decision support systems for experts. Due to its speed in terms of diagnosis and treatment, it is expected to increase the number of patients that doctors can monitor.

The remainder of this study is organized and structured as follows: Section 2 presents the details of the data that were used and introduces the proposed and selected methods. The classification performance and results of the proposed models are discussed in Section 3. In Section 4, we discuss our findings and provide directions for future research.

## 2. Methodology

**Figure 1:**
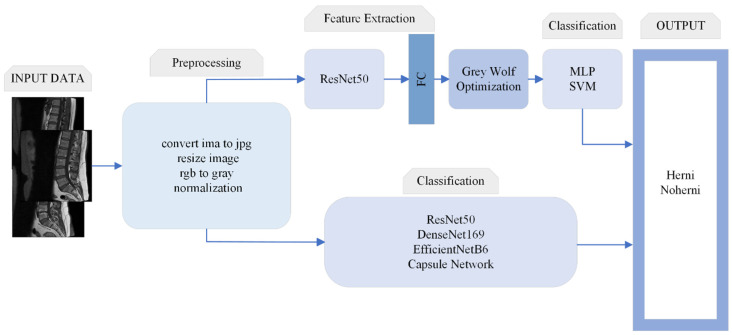
General architecture of the proposed method.

This study is designed to contribute to the clinical process in the diagnosis and treatment of diseases that can help doctors to make fast and accurate decisions. The architecture of the proposed method is shown in Figure 1.

This section describes the algorithms and basic concepts of the methods used in this study. Intervertebral disc degeneration (herniated disc) classification is performed using ResNet50, DenseNet169, EfficientNetB6, and CapsNet. In the proposed method, the GWO algorithm is used to optimize the features obtained from the fully connected layer of ResNet50, as described in further detail below.

Finally, the machine learning methods used for classification in this study—namely, the MLP and SVM algorithms—are analyzed. Table 1 below details the deep learning methods used in our study and the parameters used for classification. The given parameters are valid for all deep learning methods (ResNet50, DenseNet201, EfficientNet, CapsNet) used in this study.

### 2.1. Dataset

The dataset utilized in this study was derived from the dataset published by Sudirman et al. [21] in 2019 (https://data.mendeley.com/datasets/k57fr854j2/2; accessed on 13 October 2023), which consists of MRI slices and the corresponding radiologists’ reports containing medical information about the patients. These reports describe the findings related to the vertebral discs and provide details about the presence of hernias or other abnormalities. However, it is worth noting that the dataset has an inherent imbalance, with a significantly larger number of MRI slices showing the presence of hernias compared to those without hernias. This imbalance, combined with the limited number of non-hernia samples, was a crucial factor in the sampling process of our study.

**Figure 2:**
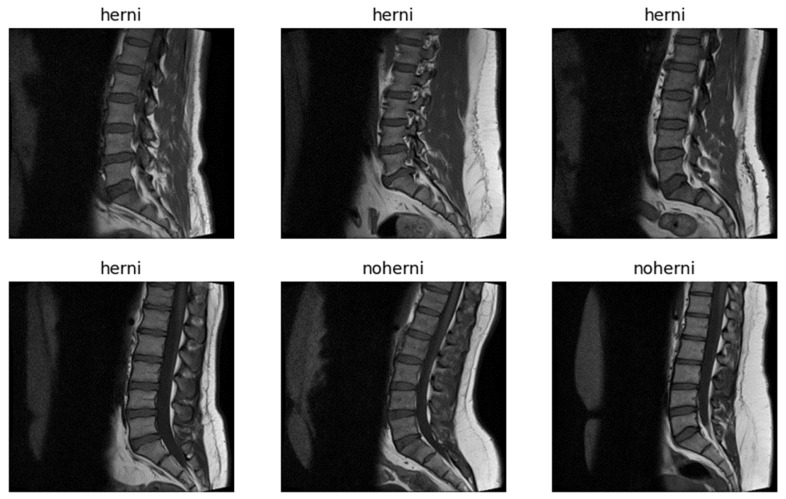
Sample images from the dataset.

The dataset originally contained 48,345 MRI slices, most of which are 320 × 320 pixels in resolution, with a few outliers at 320 × 310 pixels. These images are stored in the IMA format—a high-precision format with 12 bits per pixel—providing a level of detail greater than that of the standard 8-bit grayscale images which are typically used in most imaging datasets [21]. The higher bit depth is particularly useful for medical images, as it enables finer details that may be essential for diagnosing subtle abnormalities, such as disc herniation. Figure 2 presents randomly selected sample images from the dataset.

For the purposes of this study, a subset of 1198 selected images was used, containing both images with and without vertebral disc degeneration. The decision to reduce the dataset was driven by several factors. First, the computational resources available for processing the data were limited, preventing use of the full dataset. Second, there were undiagnosed or mislabeled samples in the dataset, which could have compromised the quality and accuracy of the model. Third, the imbalance in the data (i.e., more hernia cases than non-hernia cases) posed a challenge and, thus, careful selection was necessary to mitigate this issue. Additionally, MRI slices that appeared distorted due to errors during the scanning process were removed from the dataset to ensure that only high-quality images were included for training. From the IMA raw data, we selected T1-weighted images to achieve improved representation of brightness and darkness characteristics.

The images in the dataset, originally in IMA format, were converted to JPG for further processing. This conversion facilitated compatibility with standard image processing libraries and allowed for more efficient handling of the data. Despite the cleaning and preprocessing steps, some MRI images still showed signs of distortion, which could affect model performance. These distortions, however, reflect the realistic nature of medical imaging, where noise and imperfections are common, and can provide valuable insights into the model’s robustness. Prissilla et al. [10] adopted a similar approach to create sub-datasets from this dataset in line with certain criteria and conducted their studies on these sub-datasets.

Overall, the dataset used in this study has its challenges, such as data imbalance, the need for preprocessing, and some distortions in the images; however, it offers a valuable resource for training and testing machine learning models, especially in the domain of medical image analysis for conditions such as vertebral disc degeneration. Future work can focus on addressing the data imbalance more effectively, improving the pre- processing techniques, and expanding the dataset with additional high-quality samples to enhance the model’s generalizability and performance.

### 2.2. Pre-Processing

IMA is a raw data image format, which is usually obtained from medical imaging devices. These files contain multi-dimensional and usually grayscale images. This data in DICOM format usually contains raw pixel intensity values, which allow a specific tissue or organ to be visualized. DICOM data also contain metadata such as patient information. The intensity values contained in IMA files may not be within a specific range and, so, these values need to be adapted to the imaging system. An IMA file may contain 12-bit intensity values, as was the case for the dataset used in this study. Therefore, we first converted the files to an 8-bit image format. Then, using ready-made libraries, the images were read and converted to uint8 format. The uint8 format allows each pixel in the image to be represented as 8-bit, meaning that each pixel is an integer value between 0 and 255 (i.e., it has an 8-bit color depth). The pixel values are expressed mathematically as follows in Equation (1).

uint8 = 255 × I_norm/I_max,(1)

where I_norm is the normalized pixel value, I_max is the maximum intensity level of the pixel values (e.g., 4095 for a 12-bit image), and 255 represents the maximum pixel value in 8-bit format.

Using this formula, images with different bit depths are rescaled into the range 0–255 and each pixel can be represented using the uint8 format. Converting a JPEG format image to uint8 involves decompressing the compressed data, color space conversion, and bit depth scaling. These operations allow each pixel to be represented as an integer value in the range 0–255. After these operations, the data were divided into three sets: training, testing, and validation. These datasets were then transformed into a tensor of size n × 320 × 320 × 3. Here, n represents the number of samples in the dataset. These integer values need to be converted to float32 numbers for processing in deep learning models and, so, each pixel value was divided by 255 and scaled into the range between 0 and 1. This process allows the neural network to process image data more efficiently if they are in grayscale or color. Reducing the data to the 0–1 range reduces the likelihood of overlearning in deep learning models and simplifies computations in gradient-based optimization processes in Equation (2).

*X_norm* = *X_original*/255, (2)

where X_norm is the normalized pixel value and X_original represents the original pixel value (between 0 and 255).

The label data were then one-hot encoded, allowing each class to be represented as a vector. These data pre-processing steps are necessary for the model to learn the data more effectively.

### 2.3. Proposed Methods

In this study, a hybrid method was developed by combining several methods, including ResNet50, GWO, MLP, and SVM. As a result of training ResNet50 on the data, the required attributes were obtained from the FC layer to be used later. Afterward, using GWO, the most appropriate best_position that can be used for training with the data obtained from the FC layer was calculated. These positions were then multiplied by the attributes obtained from the FC layer and used as input data to train the intended model. The methods selected for use in this study were machine learning methods; namely, MLP and SVM.

In this section, the methods used in the study—namely, CapsNet, ResNet50, MLP, SVM, and the GWO algorithm—are detailed.

#### 2.3.1. CapsNet

CapsNet is defined in terms of structures in the brain that can learn features such as location, size, and orientation. Instead of capturing a specific feature, CapsNet is trained to find a feature and its properties in different situations. Thus, the model can understand the difference between the same capsule and object class.

CapsNet basically consists of two parts: an encoder and a decoder. While the encoder part is responsible for extracting and classifying the features of the image, the decoder part is used to reconstruct the image.

**Figure 3:**
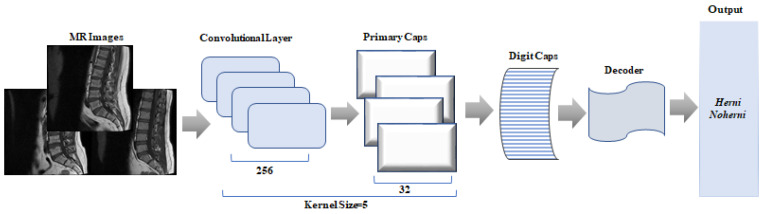
The architecture of the CapsNet model.

The encoder extracts the features of the images and converts them into vectors containing the sampling parameters. Then, training is performed using these vectors, allowing the class of a new incoming image to be predicted [20]. Similarly to the operation of variational autoencoders, the decoder attempts to reconstruct the input data of the same size using only the correctly classified capsule vector obtained at the end of training, while masking the others. The solver part of the network does not directly contribute to classification. In their work, the authors aimed to provide a regularizing effect in the classification using this part, thus creating a more stable model [22]. Figure 3 shows the CapsNet architecture.

CapsNet is a deep learning method that has become increasingly popular in recent years. However, compared to other algorithms, training CapsNets usually requires more time and computational resources. In the CapsNet model used in this study, the kernel size was set to 5 and different parameter values were tested to determine the optimal hyperparameter combination. In the training process, the learning rate was set to 0.0001, the batch size to 4, and the number of epochs to 50. The reconstruction loss coefficient was set as 4.92 and categorical cross-entropy was used as the loss function. The performance of the model was improved using these settings during the hyperparameter optimization process. Details of the parameters used are presented in Table 2 below.

#### 2.3.2. ResNet50

ResNet is a deep learning method used for classification, which has been observed to provide successful results. ResNet was introduced in 2015 by Kaiming He et al. [23], who showed that ResNet achieved impressive results in image classification tasks using the ImageNet dataset.

Transfer learning approaches leverage pre-trained model weights to address various computer vision tasks, even when resources such as datasets and computational power are constrained. In this study, we performed transfer learning on a limited medical image dataset using the pre-trained weights of the ResNet50 model, which is a CNN model consisting of 50 layers.

In studies [20,24], it has been stated that the training time of CapsNet is longer than that of traditional deep learning models, emphasizing high computational costs and long training times; this was also found to be the case in this study. For this reason, ResNet50 was used for classification training in the proposed model, as it ranked second in terms of accuracy (after the CapsNet) and can be further improved.

**Figure 4:**
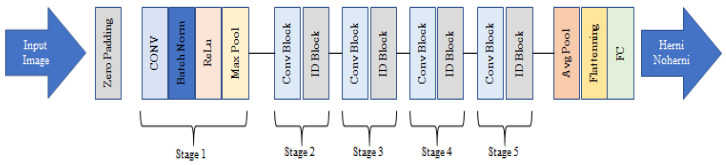
The architecture of the ResNet50 model.

ResNet50 is a ready-made function pre-trained with a 50-layer ResNet. During the training of the data as Input_shape(320,320,3) with ResNet50, the features extracted from the fully connected layer were taken (1024 for each image) and then processed further. The architecture of the ResNet50 model used in this study is presented in Figure 4 below.

**Figure 5:**
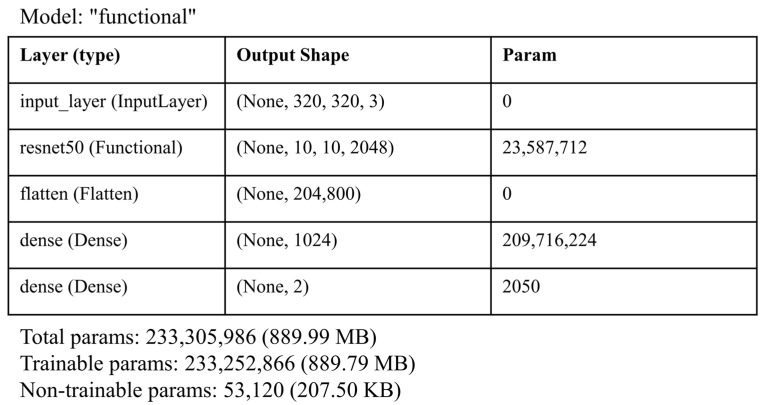
ResNet50 architecture parameters.

The architecture presented in Figure 5 represents a deep learning model based on the ResNet50 architecture, followed by additional dense layers for classification. The model’s input layer accepts images with a size of 320 × 320 pixels and 3 color channels (RGB), which is a common input size for image-based models. The first layer, ResNet50, is a pre-trained ResNet50 model that acts as the feature extractor. It outputs feature maps with a shape of (None,10,10,2048), where 2048 is the number of channels in the last convolutional layer of ResNet50. This layer has a substantial number of parameters (23,587,712), which indicates its depth and ability to capture the complex patterns in images.

The output of ResNet50 is then flattened using the flatten layer, which converts the 3D feature maps into a 1D vector of 204,800 elements. This transformation is necessary for feeding the features into the following fully connected layers, which are used for classification. The next layer, dense, is a fully connected layer with 1024 neurons, contributing 209,716,224 parameters. This layer further processes the feature vector and allows the model to learn high-level patterns in the data. Finally, the dense_1 layer is another fully connected layer with 2 neurons, corresponding to a binary classification problem. This layer produces the final output, which represents the predicted class of the input image.

In terms of the number of parameters, the model has a total of 233,510,986 parameters, indicating a highly complex architecture designed for image classification tasks. The use of the pre-trained ResNet50 as a feature extractor helps in leveraging learned representations from large datasets, such as ImageNet, which accelerates the learning process and improves the model’s ability to generalize.

#### 2.3.3. MLP

MLP is one of the algorithms chosen for classification in this study. MLPs are feed-forward neural networks that typically consist of several layers of nodes with unidirectional connections, which are usually trained via back-propagation [25,26].

The reason for using the MLP method in this study is that it is one of the leading machine learning algorithms, providing good results in classification. In this part of the model, the input_shape was set as (a,1024,1), and relu, identity, tanh, and logistic functions were used as activation functions for comparison.

ReLU, the rectified linear unit function, is defined as Reducing the data to the 0–1 range reduces the likelihood of overlearning in deep learning models and simplifies computations in gradient-based optimization processes in Equation (3).

f(x) = max(0, x).(3)

Identity, no-op activation, useful for applying a linear bottleneck, is defined as in Equation (4).

f(x) = x.(4)

Tanh, the hyperbolic tan function, is defined as in Equation (5).

f(x) = tanh(x).(5)

Logistic, the logistic sigmoid function, is defined as in Equation (6).

f(x) = 1/(1 + exp(−x)).(6)

#### 2.3.4. SVM

SVM was proposed, by Vapnik et al. [27], as a machine learning method constructed based on statistical learning theory, which was also used for classification in this study. The SVM classification process has been described in [28,29]. SVM models have been used for data optimization, classification, statistical learning, and object detection or recognition, and have been observed to provide successful results. In this study, ReLU, RBF, sigmoid, and poly functions were used as kernel functions in the SVM and the results were compared. The input shape is set as (a,1024,1), the same as for the MLP.

The linear kernel is the dot product of the input samples in Equation (7).

(7) $$K\left(x_{1},x_{2}\right)=x_{1}^{T}x_{2}.$$

The radial basis function (RBF) kernel is applied to any combination of two data points in a dataset. The dot product of the two points calculates the cosine_similarity value between both points; the higher this value, the more similar the points.

It measures the likelihood between two data points in infinite dimension and then approximates the classification based on many votes in Equation (8).

(8) $$K\left(x_{1},x_{2}\right)=\exp\left({-\gamma \left\|x_{1}-x_{2}\right\|}^{2}\right),$$

where γ is a specified parameter that must be greater than 0.

The sigmoid kernel is defined as Equation (9).

(9) $$K\left(x_{1},x_{2}\right)=\tanh\left(\gamma .x_{1}^{T}x_{2}+r\right),$$

where r is specified by coef0. In the sigmoid kernel, the similarity between two data points is calculated using the hyperbolic tangent function. The kernel function scales and possibly shifts the dot product of the two points.

The polynomial kernel changes the notion of resemblance. The kernel function is defined as Equation (10).

(10) $$K\left(x_{1},x_{2}\right)={\left(\gamma .x_{1}^{T}x_{2}+r\right)}^{d},$$

where *d* is the degree of the polynomial, γ (gamma) controls the influence of each individual training sample on the decision boundary, and *r* is the bias term (coef0) which shifts the data up or down.

#### 2.3.5. GWO

The GWO algorithm is one of the algorithms that can be used for optimization. The GWO is an optimization algorithm (metaheuristic) inspired by nature, specifically by the social behavior of grey wolf packs, which was introduced in 2014 by Seyedali Mirjalili [30]. GWO reflects the same four hierarchies as in a real grey wolf pack: α, β, δ, and ω. Of these groups, α, β, and δ are leaders, where their power is based on their rank, while ω represents subordinates. In GWO, each wolf (search agent) represents a solution. Each wolf is defined by a vector representing a solution in the problem space. Each wolf searches for a solution at a specific location. The Grey Wolf pack has a strict hierarchy; alpha wolves decide first, beta wolves assist in processing, δ wolves perform exploration, and ω wolves follow orders for decision making and exploration [31].

In this study, the GWO algorithm is used to perform feature selection for classification. The optimization function is part of the main loop of the GWO algorithm. In each iteration, it evaluates the available positions (locations) and then updates the algorithm to determine the best position.

In each iteration, the GWO algorithm updates the new positions of the alpha, beta, and delta wolves using a decreasing coefficient, where alpha, beta, and delta represent the best, second-best, and third-best positions, respectively. The new positions are combined with the average of the current positions (alpha, beta, and delta) and a random coefficient.

The fitness function calculates the classification performance of each location (feature combination). This calculated performance is obtained and measured as an accuracy score. These scores are then returned as fitness values to be used in the next iteration of the GWO algorithm.

Finally, the positions (combinations of attributes) and their fitness values are retrieved. The positions are ranked according to their fitness values, thus determining the three best positions (i.e., alpha, beta, and delta).

In this way, the GWO algorithm attempts to find the best combination of attributes for a given problem through updating the positions and evaluating the fitness values in each iteration.

The GWO algorithm uses a special formula to update the new positions using the existing positions (alpha, beta, and delta) at each iteration. The update formula is as Equation (11).

positions = (α + β + δ)/3 + a × (rand() − 0.5),(11)

where α, β, and δ represent the best, second-best, and third-best positions, respectively; a stand for the reduction coefficient, the value of which decreases depending on the number of iterations. This slowly narrows the search space; rand() generates a random number between 0 and 1.

This formula obtains new locations using a combination of the average of the existing locations and a random vector. This expands the search space and leads to better solutions.

**Figure 6:**
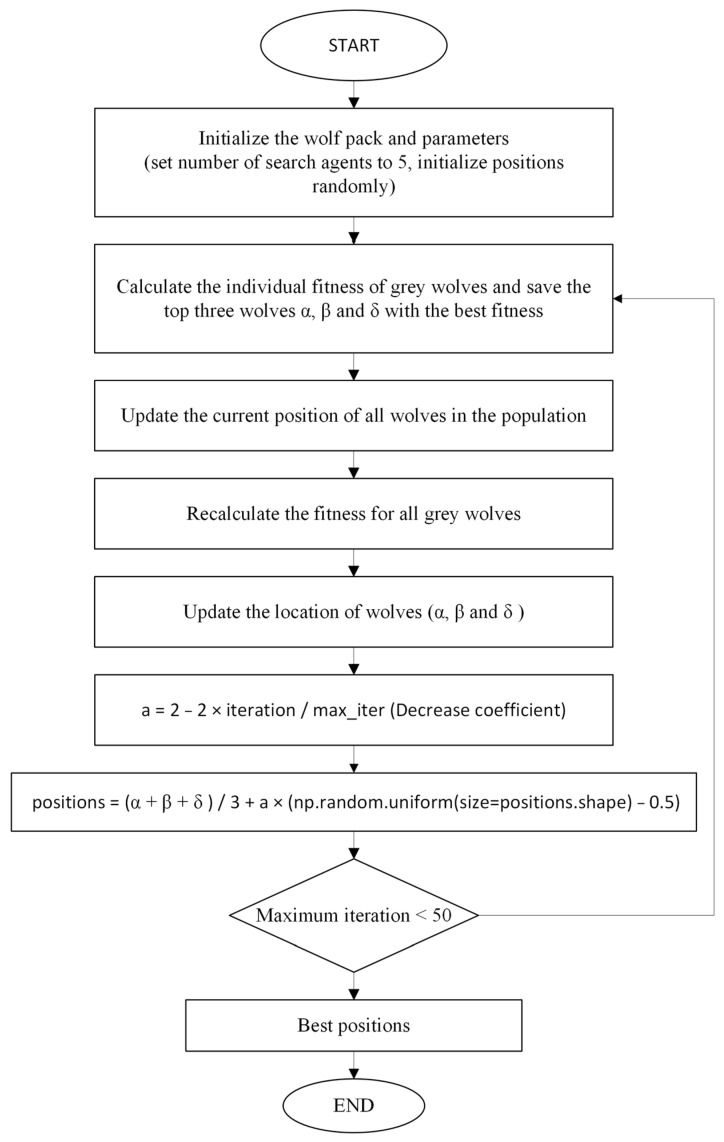
Flow chart of the proposed GWO algorithm.

In this study, GWO is used to select feature vectors to optimize the features. When we obtain the optimized weights from the GWO algorithm, after multiplying the FC features and the optimized weights, classification is performed using the result as the input to the selected methods. The flow diagram of the GWO algorithm used in this study is shown in Figure 6 below.

## 3. Experimental Results

In this section, the performance metrics used to measure the effectiveness of the methods used in the study are detailed. Then, the results of the methods used in this study, according to these performance metrics, are presented. In particular, this section describes the performance metrics of the proposed method developed for herniated disc classification.

Standard performance evaluation metrics were chosen to analyze the performance of the proposed method. These metrics are accuracy, precision, sensitivity, precision, and F1 score. The formulas for these metrics are as follows.

Accuracy: The system’s capacity to correctly decide the deterioration of the intervertebral discs.

Accuracy = (TP + TN)/(TP + TN + FN + FP).(12)

Recall: The model’s effectiveness in knowing the truth.

Recall = TP/(TP + FN).(13)

Precision: Gives the rate of correct identification of hernias.

Precision = TP/(TP + FP).(14)

F1 Score: The F1 Score is the weighted (harmonic) average of Precision and Recall values.

F1 Score = 2 × (Recall × Precision)/(Recall + Precision).(15)

In these formulas, the parameters are TP = true positive, TN = true negative, FP = false positive, and FN = false negative. In this study, these performance metrics were used to measure the performance of the applied methods.

K-fold cross-validation is a common method used to evaluate the generalization performance of a model by dividing a dataset into training and test sets. This method divides the dataset into k equal parts (folds). Then, each part is used as the test set, while the remaining k−1 parts are used as the training set. This process is repeated k times in total and the performance of the model is evaluated in each iteration. Finally, the overall performance of the model is reported by averaging the evaluation metrics obtained across all folds. This approach helps to measure the consistency of the model across different data subsets and reduces the risk of overfitting, which is why it has been used in many works. In this study, k = 5 was taken and applied for all methods. This method is also widely used for model selection and hyperparameter optimization, as a low difference between the results obtained in different folds may indicate the high generalization capacity of a model. Table 3 shows the input and output structures within each fold for all methods used in the study.

The model proposed in our study was trained on the features obtained after optimization of the output from the FC layer using GWO while training the ResNet50 architecture, which has provided good results in medical studies [32,33,34,35]. For classification, the SVM and MLP algorithms, i.e., machine learning methods, were used. Compared to alternative methods, the proposed method produced successful results. The methods and results are detailed in Table 4.

**Figure 7:**
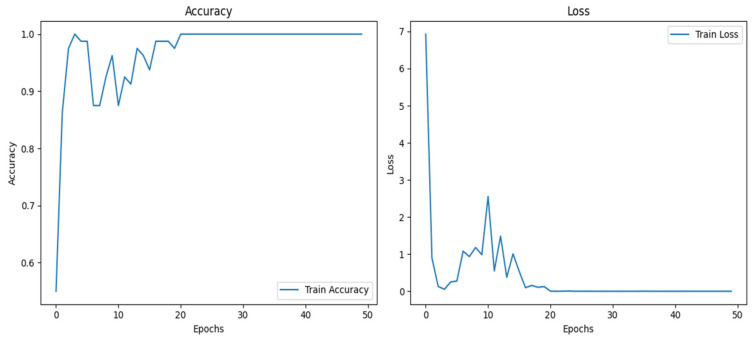
The training accuracy and loss curves of EfficientNetB6.

Table 4 compares the performance of different deep learning and machine learning methods for the considered classification problem in terms of the metrics accuracy, precision, recall, and F1 score. Among the models, CapsNet showed a high accuracy (98.41%) and a balanced performance. Meanwhile, ResNet50 lagged slightly behind, with an accuracy of 92.58%, but its performance was still strong. EfficientNetB6 showed a low accuracy (46.00%) and poor results in other metrics, compared to the other models, suggesting that this model is not suitable for the considered dataset. Although it yielded low results, the training accuracy was high in some folds. The training accuracy and loss curves in the last fold for EfficientNetB6 are shown in the Figure 7 below.

DenseNet169, on the other hand, achieved a good accuracy of 94.41% and showed a balanced performance in the other metrics.

Notably, the ResNet50-GWO-MLP and ResNet50-GWO-SVM combinations stood out as optimized methods. In particular, the ResNet50-GWO-SVM model achieved the highest accuracy of 99.42% and consistently outperformed the other models in all metrics. This suggests that the combination of methods such as GWO and SVM is effective in improving the classification performance. While the linear kernel function was used for the SVM model, ReLU was preferred as the activation function in the other methods. This difference also reflects the effects on performance.

Overall, Table 4 highlights the strengths and weaknesses of the different methods and shows that the optimized models have significant potential to achieve high performance in classification problems.

**Figure 8:**
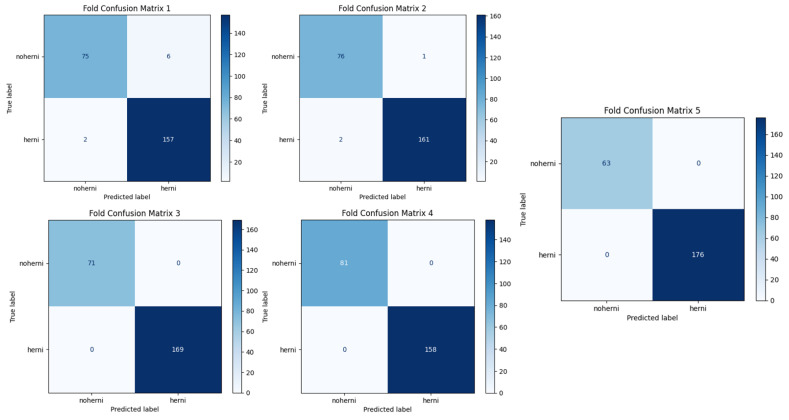
MLP with ReLu for 5 folds.

**Figure 9:**
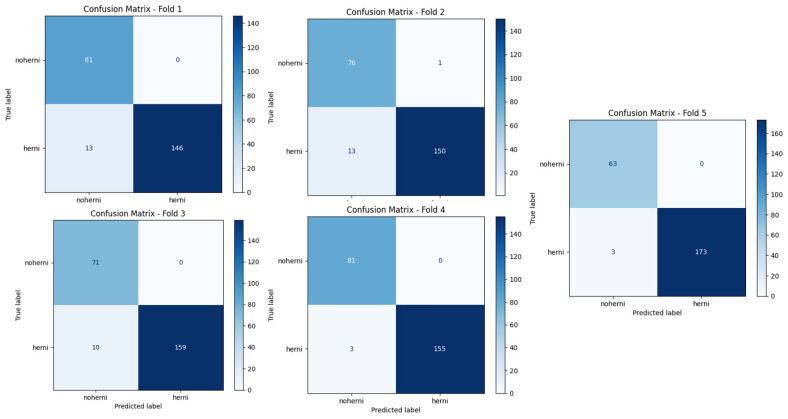
SVM with poly for 5 folds.

In terms of accuracy, ResNet50-GWO-SVM and ResNet50-GWO-MLP—that is, the methods proposed in the study—obtained the most successful results, followed by CapsNet. Figure 8 and Figure 9 show the confusion matrices of ResNet50-GWO-SVM and ResNet50-GWO-MLP, respectively.

Table 5 presents the performance metrics of the ResNet50-GWO-MLP model using different activation functions: ReLU, identity, tanh, and logistic. These metrics include accuracy, precision, recall, and F1 score, which collectively serve to evaluate the model’s performance across various dimensions.

The tanh function, despite its high recall rate, lagged behind ReLU in accuracy and F1 score as it limits the outputs between −1 and 1, making it difficult for the model to fully learn some features. The Identity function provided the lowest accuracy and F1 score values because, as a linear activation function, it has limited capacity to learn the complex relationships in the data. Therefore, ReLU offered the best overall performance, maximizing the accuracy and overall efficiency of the model, while the other functions lagged in terms of performance due to their various limitations.

The ReLU activation function achieved high performance, with an accuracy of 0.9908 and a near-perfect balance in precision (0.9909), recall (0.9908), and F1 score (0.9908). However, other activation functions—particularly identity, tanh, and logistic—slightly outperform ReLU in terms of accuracy and related metrics. Notably, the identity activation achieved the highest accuracy (0.9933) and maintained consistent precision (0.9935), recall (0.9933), and F1 score (0.9934), making it marginally better than the others.

The performance for tanh and logistic functions was also noteworthy, with both achieving identical accuracy (0.9933) and only minor differences in precision, recall, and F1 score, compared to the identity activation function. This similarity suggests that these activation functions are well-suited for the given task and dataset.

In summary, Table 5 highlights that while ReLU is a strong performer, the identity, tanh, and logistic activation functions provide a slight edge in performance for the ResNet50-GWO-MLP model. These results indicate that the choice of activation function can significantly influence model performance, and that using alternatives to ReLU, such as identity or tanh, can be beneficial in certain cases.

Activation functions cannot be used in SVM, as SVM uses the kernel trick to separate non-linear data. The kernel trick enables non-linear separations through performing inner product calculations on the space without directly moving the data into a high- dimensional space. Activation functions do not fit this structure of SVM, as they provide non-linear transformations between layers in neural networks. Therefore, for the ResNet50-GWO-SVM method, the performance metrics were calculated using different kernel functions, and the results are shown in Table 6 below.

Table 6 evaluates the performance of the ResNet50-GWO-SVM model using different kernel functions: linear, RBF, sigmoid, and polynomial (poly). The metrics considered included accuracy, precision, recall, and F1 score, which provide a comprehensive assessment of the model’s performance.

The linear kernel demonstrated the highest performance, achieving consistent metrics across all four categories, with accuracy, precision, recall, and F1 score all at 0.9942. Similarly, the RBF kernel matched the accuracy (0.9942) and recall (0.9942) of the linear kernel, while slightly surpassing it in precision (0.9943), although the difference was marginal. These results indicate that both linear and RBF kernels are highly effective for this task, with the linear kernel being computationally simpler and equally reliable.

The sigmoid kernel performed slightly poorer than the linear and RBF kernels, with an accuracy of 0.9933 and slightly reduced precision (0.9934), recall (0.9933), and F1 score (0.9933). While still effective, the sigmoid kernel presented a minor decline in performance when compared to the top-performing kernels.

On the other hand, the polynomial kernel exhibited a notable drop in performance, with an accuracy of 0.9641 and lower metrics across precision (0.9683), recall (0.9641), and F1 score (0.9647). This suggests that the polynomial kernel may not generalize well to this task or dataset, when compared to other kernel functions.

In conclusion, the linear and RBF kernels are the most suitable for the proposed model, delivering optimal performance with minimal variance across metrics. The sigmoid kernel provides moderate performance, while the polynomial kernel is the least effective for the given problem. The choice of kernel function is critical for the SVM model, with linear and RBF kernels being ideal in the considered scenario.

## 4. Discussion and Future Works

In this study, we proposed a deep learning model that utilizes ResNet50 for feature extraction, followed by fully connected layers for binary classification. The results showed that the model achieved high accuracy and robust performance, particularly when compared to traditional machine learning models, such as SVM with different kernel functions (linear, RBF, sigmoid, and polynomial). The use of ResNet50 as a pre-trained backbone allowed the model to benefit from transfer learning, leading to improved generalization and performance, even with limited labeled data. The incorporation of dense layers after ResNet50 further enhanced the model’s ability to classify complex patterns, demonstrating its potential for use in more challenging image classification tasks. Our results also indicated that the deep residual network combined with fully connected layers outperformed other architectures, including EfficientNetB6 and DenseNet169, in terms of accuracy, precision, recall, and F1 score.

The results of this study were compared with those for classification methods used in other studies, as shown in Table 7, in terms of the values obtained. Among the compared studies, 2 classes, 3 classes, and 5 classes were considered. In some studies, a very limited data set was used, which is thought to have affected the reliability of the results. The adequacy of the amount of data used in our study indicates that it can be considered as sufficient and reliable.

Table 7 presents the performance of the models used in various studies and shows that the proposed method (ResNet50-GWO-SVM) performed better than the other methods. The proposed model achieved 99.42% accuracy on a dataset of 1198 images. Among deep learning models, such as CapsNet- and CNN-based methods, InVGG [8] with 96% accuracy rate and CapsNet [12] with 96.77% accuracy rate stand out. In addition, SVM-based models developed with optimization algorithms stood out, with 93.49% [14] and 92.78% [38] accuracy rates for the BDGBA-SVM and other SVM methods, respectively.

In this study, we proposed a deep learning model that utilizes ResNet50 for feature extraction, followed by fully connected layers for binary classification. The results demonstrated that the model achieved high accuracy and robust performance, particularly when compared to traditional machine learning models such as SVM with different kernel functions (linear, RBF, sigmoid, and polynomial). The use of ResNet50 as a pre-trained backbone allowed the model to benefit from transfer learning, leading to improved generalization and performance, even when using limited labeled data. The incorporation of dense layers after ResNet50 further enhanced the model’s ability to classify complex patterns, demonstrating its potential for more challenging image classification tasks. Our results also revealed that the deep residual network combined with fully connected layers outperformed other architectures, including EfficientNetB6 and DenseNet169, in terms of accuracy, precision, recall, and F1 score.

Looking towards future work, there are several ways to further improve the model’s performance. One of the primary avenues for future development is the optimization and tuning of hyperparameters. While the current model obtained promising results, techniques such as Bayesian optimization, grid search, or random search could be employed to fine-tune parameters such as learning rates, batch sizes, and the number of neurons in the dense layers. These adjustments could lead to improved accuracy and training efficiency.

Data augmentation is another area that could be explored to further enhance the model’s robustness. Implementing additional augmentation techniques, such as random rotations, scaling, color adjustments, and flips, could help the model to generalize better, especially when training on limited datasets. Regularization methods, such as dropout, could also be incorporated to prevent overfitting and improve the model’s performance on unseen data.

While this study focused on binary classification, extending the model to multi-class classification tasks would be a promising direction for future research. Modifying the output layer and adjusting the loss function to handle multiple classes would make the model applicable in a wider range of applications, from medical imaging to object recognition. Additionally, the incorporation of attention mechanisms could be another useful enhancement. Through the use of attention layers, the model could focus on the most relevant parts of the input image, improving both accuracy and interpretability.

Finally, applying this model to real-world applications such as medical imaging, autonomous driving, and other fields requiring high-accuracy image classification could allow for practical validation of its performance. Testing the model across diverse datasets would allow for further refinement and adaptation to specific use cases, providing valuable insights into its potential for deployment in real-world scenarios.

Through exploration in these areas, the model can be further optimized and adapted, laying the foundation for more sophisticated systems in the context of image classification and related tasks.

## Figures and Tables

**Table 1:** ResNet50-GWO-MLP/SVM, ResNet50, DenseNet169, and EfficientNetB6 parameters.

Batch Size	4
Learning Rate	0.0001
Epoch:	50
Activation:	Relu
Optimizer	Adam
Loss	Binary Cross-entropy

**Table 2:** Hyperparameters used in CapsNet.

Parameter	Value
Batch Size	4
Epochs	50
Optimizer	Adam
Lam recon	4.92
Learning Rate	0.0001
Activation	Relu
Kernel Size	5
Loss	Categorical Cross-entropy

**Table 3:** Training, validation, and test inputs layers.

	Proposed Method	ResNet50 DenseNet169 EfficientNetB6	CapsNet
Input Data	(a,1024,1)	(a,320,320,3)	(a,320,320,1)
Output Data	(a,2)	(a,2)	(a,2)

**Table 4:** Comparison of results obtained with different methods.

	Accuracy	Precision	Recall	F1 Score
CapsNet	0.9808	0.9775	0.9950	0.9862
ResNet50	0.9258	0.8824	0.9258	0.8998
EfficientNetB6	0.4600	0.4353	0.4600	0.4099
DenseNet169	0.9441	0.9473	0.9441	0.9447
ResNet50-GWO-MLP	0.9908	0.9909	0.9908	0.9908
ResNet50-GWO-SVM	0.9942	0.9942	0.9942	0.9942

**Table 5:** ResNet50-GWO-MLP with different activation functions for average of 5 folds.

ResNet50-GWO-MLP	Accuracy	Precision	Recall	F1 Score
ReLU	0.9908	0.9909	0.9908	0.9908
identity	0.9933	0.9935	0.9933	0.9934
tanh	0.9933	0.9933	0.9933	0.9933
logistic	0.9933	0.9934	0.9933	0.9933

**Table 6:** ResNet50-GWO-SVM with different kernel functions for average of 5 folds.

ResNet50-GWO-SVM	Accuracy	Precision	Recall	F1 Score
linear	0.9942	0.9942	0.9942	0.9942
RBF	0.9942	0.9943	0.9942	0.9942
sigmoid	0.9933	0.9934	0.9933	0.9933
poly	0.9641	0.9683	0.9641	0.9647

**Table 7:** Comparison of the proposed method to other methods in the literature.

Reference	Method	Accuracy	Dataset
[19] (2020)	SVM	0.702	33 patients
[14] (2024)	BDGBA-SVM	0.9349	780 subjects
[12] (2023)	CNN	0.9479	515 patients
	CapsNet	0.9677	
	Proposed	0.985	
[16] (2019)	CNN-SVM	0.8040	1123 images
[36] (2022)	ResNet50	0.7747	1448 images
[37] (2021)	CNN	0.92	7948 images
[18] (2018)	MLP	0.9190	30 patients
[38] (2018)	SVM	0.9278	675 images
[10] (2023)	YOLOV5	0.7280	550 images (from same dataset)
[8] (2024)	InVGG	0.96	515 patients (from same dataset)
[9] (2019)	DenseNet201	0.88	8936 images (from same dataset)
Proposed	ResNet50-GWO-SVM	0.9942	1198 images

## Data Availability

The data presented in this study are available on request from the first author or can be downloaded from https://data.mendeley.com/datasets/k57fr854j2/2 (accessed on 28 November 2024).
